# Radiotherapy-related skin toxicity (RAREST-02): A randomized trial testing the effect of a mobile application reminding head-and-neck cancer patients to perform skin care (reminder app) on radiation dermatitis

**DOI:** 10.1186/s13063-020-04307-0

**Published:** 2020-05-25

**Authors:** Dirk Rades, Carlos Andres Narvaez, Claudia Doemer, Stefan Janssen, Denise Olbrich, Soeren Tvilsted, Antonio J. Conde-Moreno, Jon Cacicedo

**Affiliations:** 1 Department of Radiation Oncology, University of Lübeck, Lübeck, Germany; 2 Medical Practice for Radiotherapy and Radiation Oncology, Hanover, Germany; 3 Centre for Clinical Trials Lübeck, Lübeck, Germany; 4 Research Projects and Clinical Optimization, Zealand University Hospital, Koege, Denmark; 5 Department of Radiation Oncology, Hospital Universitario y Politecnico La Fe, Valencia, Spain; 6 Department of Radiation Oncology, Cruces University Hospital/ Biocruces Health Research Institute, Barakaldo, Vizcaya Spain

**Keywords:** head-and-neck cancer, radiotherapy, radiation dermatitis, oral mucositis, reminder app

## Abstract

**Background:**

Radiotherapy of head-and-neck cancer can be associated with significant toxicities including dermatitis and oral mucositis. Severe toxicities may require interruptions of the radiation treatment associated with impairment of the patients’ prognoses. This study will investigate whether the addition of a reminder app to standard care can reduce dermatitis and oral mucositis rates during radiotherapy in these patients.

**Methods:**

This randomized trial compares standard care supported by a reminder app (Arm A) to standard care alone (Arm B) with respect to grade ≥ 2 radiation dermatitis and oral mucositis at 60 Gy of radiotherapy, the minimum planned dose for patients receiving definitive or adjuvant radiotherapy for locally advanced head- and-neck cancer. Moreover, radiation-induced dermatitis and oral mucositis grade ≥ 3 at 60 Gy and both grade ≥ 2 and grade ≥ 3 at the end of radiation treatment (EOT) will be evaluated, as well as quality of life and pain. According to sample size calculations, 80 patients are required per arm within the full analysis set. Taking into account that 5% of patients will not qualify for full analysis set, 168 patients should be randomized. The impact of the reminder app will be considered clinically relevant, if the rates of grade ≥ 2 radiation dermatitis (primary endpoint) and oral mucositis (secondary endpoint) can be reduced by 20%.

**Discussion:**

If the addition of a reminder app to standard care will lead to a significant reduction of radiation dermatitis and oral mucositis, it could become a helpful tool for patients with head-and-neck cancer during radiotherapy.

**Trial registration:**

clinicaltrials.gov (NCT04110977). Registered on September 27, 2019. First patient is planned to be included in December 2019.

## Administrative information

Note: the numbers in curly brackets in this protocol refer to SPIRIT checklist item numbers. The order of the items has been modified to group similar items (see http://www.equator-network.org/reporting-guidelines/spirit-2013-statement-defining-standard-protocol-items-for-clinical-trials/).

**Table:** 

Title {1}	**Radiotherapy-related skin toxicity (RAREST-02): A randomized trial testing a reminder app to reduce radiation dermatitis in patients with head-and-neck cancer**
Trial registration {2a and 2b}.	NCT04110977, clinicaltrials.gov
Protocol version {3}	09-30-2019, version 2.0
Funding {4}	As part of the project NorDigHealth, the RAREST-02 trial was funded by the European Regional Development Fund through the Interreg Deutschland-Danmark program, reference: 087–1.1-18.
Author details {5a}	(1) Dirk Rades, Department of Radiation Oncology, University of Lübeck, Lübeck, Germany; rades.dirk@gmx.net (2) Carlos Andres Narvaez, Department of Radiation Oncology, University of Lübeck, Lübeck, Germany; carlos.narvaez@uksh.de (3) Claudia Doemer, Department of Radiation Oncology, University of Lübeck, Lübeck, Germany;claudia.doemer@uksh.de (4) Stefan Janssen, Medical Practice for Radiotherapy and Radiation Oncology, Hannover, Germany; st-janssen@gmx.net (5) Denise Olbrich, Centre for Clinical Trials Lübeck, Lübeck, Germany; olbrich@zks.uni-luebeck.de (6) Soeren Tvilsted, Research Projects and Clinical Optimization, Zealand University Hospital, Koege, Denmark; sotv@regionsjaelland.dk (7) Antonio J. Conde-Moreno, Department of Radiation Oncology, Hospital Universitario y Politecnico La Fe, Valencia, Spain; antoniojconde@gmail.com (8) Jon Cacicedo, Department of Radiation Oncology, Cruces University Hospital/ Biocruces Health Research Institute, Barakaldo, Vizcaya, Spain; JON.CACICEDOFERNANDEZBOBADILLA@osakidetza.eus
Name and contact information for the trial sponsor {5b}	**Sponsor:** University Hospital Schleswig-Holstein (UKSH), Campus Lübeck Ratzeburger Allee 160, 23,538 Lübeck, Germany **Coordinating Investigator (contact)** Prof. Dr. Dirk Rades Department of Radiation Oncology University of Lübeck Ratzeburger Allee 160 23,538 Lübeck, Germany. Tel.: + 49-(0)451–500-45,400 Fax: + 49-(0)451–500-45,404 Email: Rades.Dirk@gmx.net
Role of sponsor {5c}	The sponsor and the funding body have no role in the design of the study, in collection, analysis and interpretation of the data and in the writing of the manuscript.

## Introduction

### Background and rationale {6a}

Many patients with squamous cell carcinoma of the head and neck (SCCHN), particularly those patients with locally advanced disease, receive radiotherapy. If radiotherapy is administered as definitive treatment (i.e*.*, without preceding surgery), it is generally combined with chemotherapy [1]. In an adjuvant situation (i.e*.*,following surgery), concurrent chemotherapy will be administered if risk factors (incomplete resection and/or extracapsular [ECS] spread of lymph nodes metastases) exist.

Radiotherapy of SCCHN can be associated with significant toxicities including dermatitis and oral mucositis. Severe toxicities may require interruptions of the radiotherapy series that can impair the prognoses of these patients [2, 3]. To avoid severe (grade ≥ 3) radiation toxicities, it is important to avoid or postpone grade 2 toxicities. Grade ≥ 2 dermatitis and grade ≥ 2 mucositis rates were very high in previous studies (86–92% and 86–100%, respectively) and require improvement [4–6]. In the previous RAREST-01 trial that compared the dressing Mepitel® Film to standard skin care in patients irradiated for head-and-neck cancer, dermatitis rates were lower than expected in both groups [7, 8]. In the RAREST-01 trial, standard skin care was supposed to be performed four times daily, which required a high level of discipline from the patients. Daily reminders by medical staff members regarding the importance of skin care likely improved the patients’ compliance resulting in less radiation dermatitis. It may be questioned whether the daily reminders by staff members can be replaced by a mobile application (a reminder app).

### Objectives {7}

This study aims to show that standard skin care supported by a reminder app is superior to standard skin care alone regarding the avoidance of grade ≥ 2 dermatitis up to 60 Gy in patients irradiated for locally advanced head-and-neck cancers. The null hypothesis of equal grade ≥ 2 dermatitis rates in both groups is tested against the two-sided alternative hypothesis of different dermatitis rates.

### Trial design {8}

This is a randomized, active-controlled, parallel-group trial, which will primarily compare two treatments of radiation dermatitis in patients with head-and-neck cancer: standard care supported by a reminder app (Arm A) versus standard care alone (Arm B).

## Methods: participants, interventions and outcomes

### Study setting {9}

The study will be performed in Germany (one university hospital, one private practice) and Spain (two university hospitals). Study sites can be obtained at clinicaltrials.gov.

### Eligibility criteria {10}

Inclusion criteria

1. Histologically proven locally advanced SCCHN
2. Indication for radiotherapy or radio-chemotherapy
3. Possession of and ability to use a smartphone
4. Age ≥ 18 years
5. Written informed consent
6. Capacity of the patient to contract

Exclusion criteria

1. Nasopharynx cancer
2. Pregnancy, lactation
3. Treatment with epidermal growth factor receptor antibodies (given or planned)
4. Expected noncompliance

Radiation dermatitis and oral mucositis will be assessed by two independent observers (specially trained nurses, technicians, or physicians) weekly during the period of radiotherapy, at 60 Gy and at end of treatment (EOT). If the graduation varies between the two observers, toxicities will be assessed by an additional observer. Observers are required to be very experienced in rating toxicities and will additionally undergo a particular briefing prior to the start of this study.

### Who will take informed consent? {26a}

Informed consent will be taken by specially trained physicians registered as investigators for this trial.

### Additional consent provisions for collection and use of participant data and biological specimens {26b}

Not applicable.

## Interventions

### Explanation for the choice of comparators {6b}

In a previous trial, daily reminders by medical staff members regarding the importance of skin care likely improved the patients’ compliance resulting in less radiation dermatitis [7, 8]. It may be questioned whether the daily reminders by staff members can be replaced by a mobile application (reminder app).

### Intervention description {11a}

#### Standard skin care

In both the experimental (A) and the control arm (B), standard skin care is to be performed by the patient from the beginning of radiotherapy. Standard skin care may vary at the participating centers. At the University of Lübeck, it includes fatty cream with 2–5% urea (fatty cream alone, if patients do not tolerate urea) and mometasone furoate cream. The treatment will be continued until 1 week after EOT or until a patient experiences grade ≥ 2 moist desquamation or grade ≥ 3 radiation dermatitis.

#### Standard mouth care

In both arms, standard mouth care is to be performed by the patient from the beginning of radiotherapy to prevent or at least postpone radiation-induced oral mucositis. At the University of Lübeck, it initially consists of an antibacterial mouth rinse to be used four times a day. If the patient experiences pain, lidocaine hydrochloride + plus dexpanthenole solution will be added. Alternatively, benzdyamine hydrochloride solution may be used. In case of an oral edema/swelling, hydrocortisone acetate may be used.

#### Reminder app

The patients in the experimental arm (Arm A) are supported by a reminder app (“CareReminder”), which has been developed by the professional company Nextlabel OHG from Lübeck, Germany. This app will remind the patients four times a day to perform skin and mouth care. Instructions are given how to perform it properly. The patients may postpone each required care procedure for up to 2 hours. Finally, the patients are asked to state for each procedure whether or not they performed it. To increase the patients’ motivation, they will earn points for each successfully performed procedure.

### Criteria for discontinuing or modifying allocated interventions {11b}

In case of grade ≥ 2 moist desquamation or grade ≥ 3 radiation dermatitis, each day antiseptic agents will be administered for wound cleansing followed by administration of silicon or calcium alginate bandage. This treatment will be continued until moist desquamation radiation disappears and radiation dermatitis improves to grade 2. If local anesthetics (lidocaine, benzdyamine) are not sufficient in reducing pain due to oral mucositis, systemic analgesics are given, usually metamizole and if required morphine derivatives.

### Strategies to improve adherence to interventions {11c}

Not applicable.

### Relevant concomitant care permitted or prohibited during the trial {11d}

 If required, any type of concomitant care and interventions are permitted during the trial for treatment of other radiotherapy- or radio-chemotherapy-related toxicities and comorbidities not related to radiotherapy or radio-chemotherapy.

### Provisions for post-trial care {30}

Following EOT (i.e., end of study), the patients receive the standard follow-up program for patients with head- and-neck cancer. Harm from trial participation is not expected, since all participating patients receive the same anticancer treatment as they would have received if not participating.

### Outcomes {12}

Primary endpoint is the rate of grade ≥ 2 radiation dermatitis Common Terminology Criteria for Adverse Events (CTCAE v5.0) at 60 Gy of radiotherapy, the minimum planned total dose for all patients receiving definitive or adjuvant radiotherapy for locally advanced head and neck cancer. In addition, the following endpoints will be evaluated:

1. Radiation dermatitis grade ≥ 2 at EOT
2. Radiation dermatitis grade ≥ 3 at 60 Gy and at EOT
3. Radiation-induced oral mucositis grade ≥ 2 at 60 Gy and at EOT
4. Radiation-induced oral mucositis grade ≥ 3 at 60 Gy and at EOT
5. Quality of life
6. Pain

### Participant timeline {13}

A total of approximately four contributing centers are planning to participate, which aim to include an average of 21 patients per year. The recruitment of all 168 patients should be completed within 24 months. The treatment period will be 6–7 weeks. This equals a total running time for the trial of 26 months.

### Sample size {14}

This study aims to show that standard skin care supported by a reminder app is superior to standard skin care alone regarding the avoidance of grade ≥ 2 dermatitis up to 60 Gy in patients irradiated for locally advanced head-and-neck cancers.

The null hypothesis of equal grade ≥ 2 dermatitis rates in both groups is tested against the two-sided alternative hypothesis of unequal dermatitis rates. The calculation of the sample size considered the following assumptions:

- Application of a chi-square test
- Two-sided significance level of 5%
- Rates of grade ≥ 2 dermatitis of 86–92% with standard care alone according to previous studies
- Assumption of a grade ≥ 2 dermatitis rate of 85% in the standard care alone group (“worst-case”)
- Clinical importance of the impact of the reminder app = reduction of grade ≥ 2 dermatitis by 20%
- Statistical power of 80%

When considering these assumptions, 80 patients are needed per arm within the full analysis set. Assuming that 5% of the recruited patients will not qualify for the full analysis set, 168 patients need to be randomized.

### Recruitment {15}

A total of approximately four contributing centers are planning to participate, which aim to include an average of 21 patients per year. The recruitment of all 168 patients should be completed within 24 months.

## Assignment of interventions: allocation

### Sequence generation {16a}

The patients will be assigned two code numbers: the number of the contributing center plus a patient identity number, continuously ascending, starting with 001.

After registration, patients will be randomized in a 1:1 ratio to receive either standard care supported by a reminder app (Arm A) or standard care (Arm B) for treatment of radiation-related skin toxicity.

A stratified block randomization will be performed. Stratification will be done using the following prognostic factors:

1. Tumor site: oropharynx/oral cavity versus hypopharynx/larynx.
2. Treatment approach: radio-chemotherapy versus radiotherapy alone.
3. Participating site.

The randomization will be performed centrally at the ‘Zentrum für Klinische Studien’ (ZKS) at the University of Lübeck, Germany via fax. The proceeding is based on standard operating procedures (SOPs) of the ZKS. The fax document has to be completed and finally be signed and dated by the investigator. Once the randomization is allocated to the patient it cannot be changed.

### Concealment mechanism {16b}

Not applicable.

### Implementation {16c}

Generation of the allocation sequence, enrollment of participants and assignment of participants to interventions will be performed by specially trained physicians registered as investigators for this trial.

## Assignment of interventions: blinding

### Who will be blinded {17a}

Data analysts and statisticians will be blinded.

### Procedure for unblinding if needed {17b}

Not applicable.

## Data collection and management

### Plans for assessment and collection of outcomes {18a}

The following parameters will be recorded prior to radiotherapy: Demographics, medical history, concomitant diseases, physical examination, performance status, primary tumor site, stage and histology, upfront surgery, planned chemotherapy, status of skin and oral mucosa, pain, and quality of life.

The following parameters will be assessed throughout the course of the trial:

1. Radiation dermatitis: assessed by two observers weekly during radiotherapy, at 60 Gy and at EOT according to CTCAE v5.0 [9]. If graduation varies, a third observer will be involved.
2. Oral mucositis: assessed by two observers weekly during radiotherapy, at 60 Gy and at EOT according to RTOG criteria [10–13]. If graduation varies, a third observer will be involved.
3. Quality of life: assessed prior to radiotherapy, at 60 Gy and at EOT with European Organisation for Research and Treatment of Cancer (EORTC) Quality of Life Questionnaire (QLQ)-C30 Version 3.0 and EORTC QLQ-H&N35.
4. Pain (within radiation fields): assessed prior to radiotherapy, weekly during radiotherapy, at 60 Gy and at EOT with a visual analog scale (from 0 = no to 10 = maximum pain).
5. Other adverse events will be assessed on an ongoing basis according to CTCAE v5.0 [9].

**Fig. 1:**
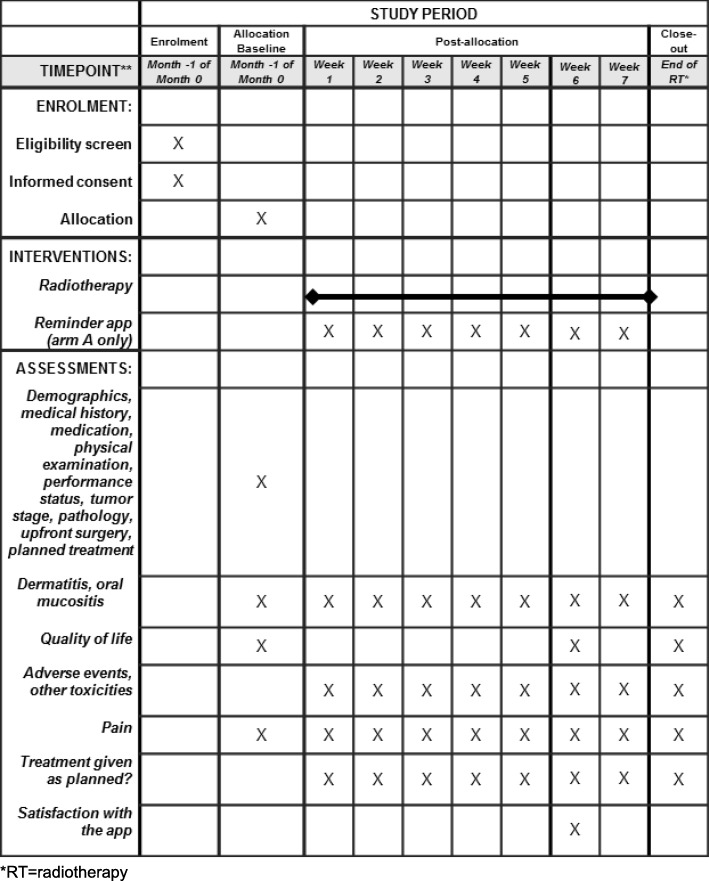
Schedule of enrolment, interventions and assessments. *RT radiotherapy

The timeline of the study procedures including the assessments is given in Fig. 1.

### Plans to promote participant retention and complete follow-up {18b}

The last day of radiotherapy (EOT) is also the end of the follow-up period. Patients are seen at least 5 days per week by medical staff members. Thus, it is unlikely that patients will be lost to follow-up. If patients withdraw their consent to participate in the trial or die during the course of radiotherapy, the data available until this point in time will be used for analyses.

### Data management {19}

#### Patient identification list

All data relating to patients will be recorded in a pseudonymous way. Each patient will be identifiable only by the unique patient number, date of birth and sex. A patient identification list will only be kept in the relevant trial centers and will not be forwarded to the sponsor.

#### Documentation sheet

Data collection will be done using the data documentation sheets**.**

The data documentation sheets must be filled in using a ballpoint pen. Fountain pens or pencils may not be used. Corrections must be made as follows: cross the error out once with a straight line, enter the correct information next to it and note the date and/or reason for correction. Comments must be made if data fields cannot be filled in because of missing information.

The sheets should be filled in as soon as possible and should be submitted to the checker for review, signed, dated and forwarded to the trial management. All data will be pseudonymized before they are forwarded for analysis. The data will be handled according to the General Data Protection Regulation (GDPR). The data will be brought into one database and analyzed in accordance with a predefined statistical analysis plan. At this stage, subgroup analyses are not planned. At each interim analysis, the need for subgroup analyses will be re-evaluated.

#### Storage of trial documents

The originals of all key trial documents, including the documentation sheets, will be kept at the trial headquarters (i.e., the sponsor responsible for the trial) for a minimum of 10 years after the final report.

The principal investigator/head of the trial center will keep all administrative documents (written correspondence with the ethics committee, regulatory authorities, trial management, trial headquarters), patient identification list, signed informed consent forms, copies of the documentation sheets and general trial documentation (protocol, amendments) for the abovementioned period. Original patient data (patient files) must also be kept for the length of time stipulated for the trial centers, but not for less than 10 years.

### Confidentiality {27}

Data will be collected in accordance with the regulations set out in the Data Protection Act. All findings from the clinical trial will be stored on electronic data storage devices and treated with utmost confidentiality. Organization measures have been taken in order to prevent the data from being communicated to unauthorized persons. Patients will only be identified via their individual patient numbers throughout the entire documentation and evaluation phase and their full name will not be used.

### Plans for collection, laboratory evaluation, and storage of biological specimens for genetic or molecular analysis in this trial/future use {33}

Not applicable.

## Statistical methods

### Statistical methods for primary and secondary outcomes {20a}

All data recorded in the case report forms describing the study population, toxicity and quality of life will be analyzed descriptively. Categorical data will be presented in contingency tables with frequencies and percentages. Continuous data will be summarized with at least the following: frequency (n), median, quartiles, mean, standard deviation (standard error), minimum and maximum. Number of patients with protocol deviations during the study and listings describing the deviations will be provided. In general, chi-square tests will be used to compare percentages in a two-by-two contingency table, replaced by Fisher exact test if the expected frequency in at least one cell of the associated table is less than five. Stratified two-by-two contingency tables will be analyzed using Cochran-Mantel-Haenszel tests. Logistic regression models serve as multivariable methods for binary endpoint data. Comparison of ordinal variables between treatment arms will be performed using the asymptotic Wilcoxon-Mann-Whitney test, replaced by its exact version in case of ordinal categories with small number of categories and/or sparse data within categories. Any shift in location of quantitative variables between study groups will be performed with the Wilcoxon-Mann-Whitney tests as well. Time-to-event data will be analyzed by Kaplan-Meier methods, when merely non-informative censoring occurs. For statistical comparison, the log rank-test will be provided supplemented by multivariate Cox proportional hazards models. The data analysis will be performed according to the statistical analysis plan, and which will be finalized prior to database lock and prior to any statistical analysis.

### Interim analyses {21b}

After termination of the radiation treatment of one third (N = 56) and two thirds (N = 112), respectively, of the patients, interim analyses will be performed. Recruitment will be put on hold until the 56 and 112 patients, respectively, have completed their radiation treatment. In case of a dissatisfaction rate > 25%, the reminder app needs to be modified and adapted. In case of a dissatisfaction rate > 50%, the study will be terminated prematurely.

### Methods for additional analyses (e.g., subgroup analyses) {20b}

Not applicable.

### Methods in analysis to handle protocol nonadherence and any statistical methods to handle missing data {20c}

The full analysis set includes all randomized patients who have started either therapy with Arm A or with Arm B. The full analysis set will be analyzed according to the intent-to-treat principle, i.e., patients will be analyzed in their initial group of randomization.

The per protocol set will comprise all patients of the full analysis set and will exclude patients if any of the following criteria are met:

- Administration of less than 60 Gy if the reason for discontinuation was any other than death or unacceptable toxicity
- Delay of radiotherapy for more than 7 days prior to reaching 60 Gy

All patients in the per protocol set will be analyzed within their group of actual treatment received.

### Plans to give access to the full protocol, participant level-data and statistical code {31c}

Not applicable.

## Oversight and monitoring

### Composition of the coordinating center and trial steering committee {5d}

Not applicable.

### Composition of the data monitoring committee, its role and reporting structure {21a}

A data monitoring committee is not needed, since all patients participating in this trial receive the same anticancer treatment, the same number and type of visits, and the same treatment for radiation dermatitis, oral mucositis and other toxicities as they would have received if not participating in the trial.

### Adverse event reporting and harms {22}

#### Assessment and documentation of adverse events

The severity of adverse events should be assessed using the national (CTCAE, version 5.0 [9]. Oral mucositis will be assessed according to the criteria of the Radiation Therapy Oncology Group (RTOG) [10–13].

Otherwise, the 5-point scale below will be used to describe an adverse event.

Scale: 1 = mild, 2 = moderate, 3 = severe, 4 = life-threatening and 5 = fatal.

The following scale should be used to describe the likelihood that the event was caused by the trial treatment:

1 = certain/definite, 2 = probable, 3 = possible, 4 = unlikely, 5 = not related, 6 = not assessable.

#### Reporting of serious adverse events and unexpected adverse events

Serious adverse events and unexpected adverse events must be reported within 24 hours after their detection/onset by fax to the coordinating investigator.

### Frequency and plans for auditing trial conduct {23}

The ZKS Lübeck will conduct clinical on-site monitoring at the German sites according to Good Clinical Practice (GCP) and written SOPs to ensure the patients’ rights and safety as well as the reliability of trial results. For initiation, trial sites will be visited on-site by a clinical research associate of the ZKS Lübeck. During the trial, sites will be visited at regular intervals depending on the rate of recruiting and data quality. Informed consent and defined key data will be checked of all patients. The medical file of each patient will be screened for adverse and serious adverse events. Patients’ questionnaires will be checked for their existence. According to SOPs, all trial-specific monitoring activities will be defined before starting the trial and documented in writing (monitoring manual). The sites in other countries will be monitored according to the corresponding national regulations. No regular audits are planned. However, to ensure correct execution of the study, audits may be conducted if necessary. As the current study is not linked to any German pharmaceutical or medicinal product act, no inspections of higher federal authorities are scheduled.

### Plans for communicating important protocol amendments to relevant parties (e.g. trial participants, ethical committees) {25}

Amendments to the study protocol may only be implemented if approved again by the ethics committee responsible. Only the coordinating principal investigator may carry out such changes. However, all co-investigators should contact the coordinating principal investigator if modifications seem to be necessary. In case of changes to the study protocol, all investigators will be informed after ethics committee approval and the notice will have to be confirmed.

## Dissemination plans {31a}

The coordinating principal investigator will work toward comprehensive internal and external dissemination of project results and knowledge. Coordinating principal investigators, biostatisticians, and reference centers will create a report according to the CONSORT statement regardless of regular or abnormal study termination. The scientific results will be published in international, peer-reviewed journals of the highest possible quality. In addition, results will be presented at major medical congresses and symposia.

Due to methodological and statistical aspects, results will be published only after study database closure. All reports and publications related to the study need to be coordinated with the biostatistician to avoid misinterpretation of statistical results. Conclusions need to be statistically secured and require approval of the statistician. The local centers are entitled to use the recorded data for additional scientific exploitation under their own name, but not before the main results have been published. There are no exceptions to this rule. Any sub-publication requires approval by the coordinating principal investigator. For publications of any kind the study acronym RAREST-02 will be used.

## Discussion

Many patients who receive radiotherapy or radio-chemotherapy for locally advanced SCCHN experience radiation-related acute toxicities including dermatitis and oral mucositis. If these toxicities become severe, an interruption of the radiotherapy treatment may be required. Interruptions of 1 week or longer were reported to impair the patient’s prognosis. In a retrospective study of patients irradiated for SCCHN, local control (*p* = 0.015) and survival (*p* = 0.021) were significantly worse with such an interruption of the radiation treatment [2]. To avoid toxicity-related interruptions of radiotherapy, it is reasonable to avoid or at least postpone grade 2 reactions. This accounts for both radiation dermatitis and radiation-related oral mucositis. In previous studies of radio-(chemo)-therapy for SCCHN, rates of grade ≥ 2 radiation dermatitis and grade ≥ 2 oral mucositis were reported to range from 86% to 92% and from 86% to 100%, respectively, although the patients had received standard skin and mouth care [1, 4–6].

Since the administration of an absorbent, self-adhesive dressing appeared a promising approach to reduce radiation dermatitis, we performed a randomized trial (RAREST-01) that compared the new dressing Mepitel® Film to standard skin care with respect to radiation dermatitis rates [7, 8]. However, almost half of the patients did not tolerate the dressing, and the trial had to be stopped prematurely. In those patients already included, the new dressing appeared to be not superior to standard skin care. Surprisingly, the radiation dermatitis rates were lower than expected in both groups. One possible explanation for this unexpected finding could be that the patients were reminded daily by staff members to perform their skin care, which generally required a high level of discipline and compliance. Improved compliance of patients resulting in a proper performance of daily skin care might have led to a lower incidence and severity of radiation dermatitis. One question is whether the daily reminders by the staff members can also be successfully done by a specific reminder app. Therefore, in this trial the effect of a reminder app on radiation dermatitis rates and, additionally, on oral mucositis rates is being investigated. If the addition of such a reminder app to standard care leads to less radiation dermatitis and oral mucositis, it could become a helpful tool for patients irradiated for head-and-neck cancers.

## Trial status

Protocol version 2.0 from 09-30-2019, recruitment is planned to start in December 2019 and will be completed within 24 months.

## Data Availability

Not applicable, as no datasets were generated or analyzed during the current study so far. The study has been registered at clinicaltrials.gov (identifier: NCT04110977), where details of the study protocol presented in this manuscript are available.

## Abbreviations

CTCAE: Common Terminology Criteria for Adverse Events
ECS: extracapsular spread
EORTC: European Organisation for Research and Treatment of Cancer
EOT: end of radiation treatment
GCP: Good Clinical Practice
QLQ: Quality of Life Questionnaire
RTOG: Radiation Therapy Oncology Group
SCCHN: squamous cell carcinoma of the head and neck
SOP: standard operating procedure
